# General- and Oral-Health-Related Predisposing Factors for Interrupting Military Service in the Finnish Defence Forces

**DOI:** 10.1093/milmed/usab311

**Published:** 2021-07-24

**Authors:** Pertti Patinen, Tarja Tanner, Jesse Honkanen, Leo Tjäderhane, Jari Päkkilä, Vuokko Anttonen, Antti Kämppi

**Affiliations:** Finnish Defence Forces, Centre for Military Medicine, Riihimäki 11311, Finland; Research Unit of Oral Health Sciences, University of Oulu, Oulu 90220, Finland; Institute of Dentistry, University of Eastern Finland, Kuopio 70211, Finland; Research Unit of Oral Health Sciences, University of Oulu, Oulu 90220, Finland; Department of Oral and Maxillofacial Diseases, University of Helsinki, and Helsinki University Hospital, Helsinki 00014, Finland; HUS, Helsinki University Hospital, Helsinki, Finland; Research Unit of Mathematical Sciences, University of Oulu, Oulu 90014, Finland; Research Unit of Oral Health Sciences, University of Oulu, Oulu 90220, Finland; Department of Oral and Maxillofacial Diseases, University of Helsinki, and Helsinki University Hospital, Helsinki 00014, Finland

## Abstract

**Introduction:**

Finland’s security policy relies heavily on its own independent national defense, which is based on conscription. In 2011, 26,492 conscripts started their military service in Finland. Of these, 1,706 interrupted their military service and 191 changed to civilian service. Conscripts who interrupt their service seem to have an increased tendency to smoking, alcohol consumption, and taking snuff, which previous studies suggest to have strong associations with the need for restorative dental treatment and with lower socioeconomic status. The aim of this study was to compare the general and oral health habits between Finnish conscripts who interrupt their service and those who completed their military service and to find out what general- and oral-health-related factors could be used in predicting interruption of service.

**Methods:**

The study population consisted of 13,819 conscripts taking an oral examination during the service. Of these, 8,449 answered a computer-based anamnestic questionnaire and 264 interrupted their service.

Predisposing factors on the anamnestic questionnaire for interrupting military service were evaluated by using a binary logistic regression model. The statistically significant factors were selected to form a sum variable which finally consisted of seven predisposing questions. Odds ratio (OR) values and 95% confidence intervals were calculated for each question and for the sum variable. Predictive accuracy was assessed by area under the receiver-operating curve.

**Results:**

The most obvious predisposing factor among those who interrupted their service compared to the reference group was lack of weekly physical exercise (OR = 5.80). The risk for interruption of military service was 68.6 times higher in cases where a subject exhibited six predisposing factors out of seven compared to those who had none.

**Conclusion:**

As a conclusion, a set of statistically chosen anamnestic questions could help identify conscripts who have an increased risk of interruption of military service in addition to a risk of dental problems.

## INTRODUCTION

Conscription mandates military service lasting from 5.5 to 12 months for all young men in Finland. Since 1996, it has been also possible for women on a voluntary basis. For either ethical or religious reasons, about 1.5% of each age cohort are allowed to do 12 months service in civilian institutes. More than 30,000 young males are legally ordered to undertake military service each year and around 400 females enter the service voluntarily. All undergo a pre-military health examination by civilian doctors. After the examination, about 26,500 are finally ordered to start their military service.^1,2^ About 1,700 individuals interrupt their service during the first 3 weeks. Finally, roughly 20,000 complete the service each year, thus constituting about 76% of each age cohort. Almost all discharges are for health reasons.^2^ Since all localities, levels of education, and socioeconomic backgrounds are represented by the conscripts, reasonable nationwide deductions on the general and oral health of young healthy people can be made.

Conscripts who interrupt their military service for medical reasons must complete their service before the age of 28 years. They are usually given a 6-48 months recovery time during which they have their medical problems treated. If a conscript still has a medical impediment at the age of 28 years, he will be released from military service. This decision is always made based on a medical or mental disability diagnosis.

During 2011, 26,492 conscripts started their military service in Finland. Of those, 1,706 interrupted their service and 191 changed to civilian service after starting normal military service. Conscripts interrupt their service most often during their first 3 weeks of conscription,^3^ with the most common reasons for the interruption being mental and behavioral disorders (30.8%); musculoskeletal and connective tissue disorders (19.6%); respiratory diseases (11.2%); and endocrine, nutritional, and metabolic disorders (8.2%).^3^ Oral-related factors, however, were not included in a recent study. Interruption of military service has been studied,^4^ but research into interrupted conscripts and oral-health-related factors is sparse.

Oral health and restorative treatment need among conscripts have already been studied four times in Finland. The studies were conducted in a very similar set-up between 1979 and 2015.^5–8^ Kämppi and Tanner^7,8^ extended their study field to the dental restorative treatment need, focusing more extensively on sociodemographic factors related to restorative treatment need. Smoking, alcohol use, taking snuff, and other risky oral-health-related behavior habits have strong associations with the need for dental restorative treatment and lower socioeconomic status.^9,10^

### Aim

The aim of this study was to compare the general and oral health habits of Finnish conscripts who interrupt their service with those who continued their military service in 2011. A further aim was to find out what general- and oral-health-related factors could be used in predicting interrupted military service among the young Finnish male population.

### Hypothesis

The main hypothesis of this study is that well-chosen self-reported information and a need for restorative treatment in an oral examination could predict interruption of military service in the Finnish Defence forces.

## MATERIALS AND METHODS

**FIGURE 1.**
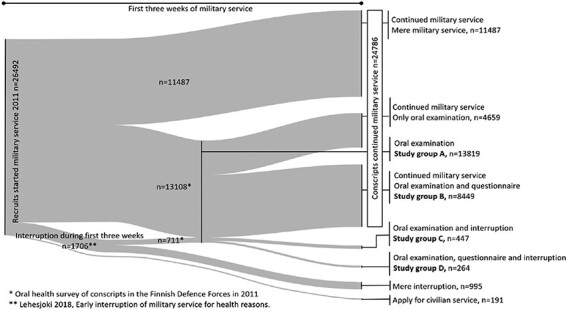
A Sankey diagram of conscripts who started military service in 2011 and their path after the first 3 weeks in military service.

The study population comprised the participants in the Oral Health of the Finnish Conscripts 2011 study.^7,8^ The data from that project were combined with the data on those who interrupted their military service.^3^ The participants were categorized by whether they completed the service and participated in the oral examination and the original oral health questionnaire (Groups A-D, Fig. 1).

Study groups C and D were formed on whether the oral examination was done and the questionnaire answered among those who interrupted the service. Study groups C and D were compared to either control study groups B (those with the oral examination and questionnaire and continued service) or A (those who responded to the questionnaire and continued service) accordingly (Fig. 1).

### The Oral Health of the Finnish Conscripts 2011

The Oral Health of the Finnish Conscripts 2011 epidemiological cross-sectional study was carried out in the health centers of 20 garrisons (of a total 24) of the Finnish Defence forces in January and July 2011. Four garrisons were excluded from the study because of the limited personnel in dental services. In five biggest garrisons every fifth recruit were selected in alphabetical order to the study. Random sampling was inbuilt. Recruits were placed in rooms according to their surname regardless of any socioeconomic, demographic, or geographic factors. In smaller garrisons all recruits were selected to the study. There were no refusals.

The original study population consisted of 13,564 men and 255 women born in 1990, 1991, or 1992 (mean age 19.6 years). The oral health examination was conducted in connection with a mandatory health examination. If in oral examination caries reached the dentin, bitewings were taken, and status was complemented accordingly. In addition, all the conscripts had the opportunity to respond to a computer-assisted anamnestic questionnaire that examined individual backgrounds and oral health habits. Altogether, 8,713 conscripts answered the questionnaire (Fig. 1, groups B and D).^7,8^ In the oral examination, the restorative treatment need at tooth level of each conscript was recorded according to the 1997 criteria for epidemiological studies by the World Health Organization^11^ and the Defence forces protocol was followed^12^ (Fig. 1, group A). The findings were recorded by a dental assistant. Dental units at the garrison dental clinics were used for the examinations. The dental unit light was used for the examination as well as a probe and an oral mirror. Oral examination data were recorded on the Mildoc patient information system of the Defence forces.

### Anamnestic Questionnaire

The original questionnaire consisted of 50 questions.^7,8,13^ All the questions were carefully considered and the final selection was made by authors.

### The Selection of the Questions Used in the Study

Before the final decision on which questions should be included, binary logistic pretests were performed. Questions concerning alcohol; smoking; taking snuff; fathers’, mothers’, and one’s own level of education; conscript entry group (winter or summer); the number of children; and dating status were statistically pretested together with the questions chosen for this study. The possibility of collinearity among the factors in the final model (Table III) was checked by calculating variance inflation and generalized variance inflation factors. All values were small, less than 1.5. The final model was reached by gradually adding explanators to the model. After the pretests, the variables included were physical exercise, working status, one’s own perception of how the state of dentition affects well-being, use of xylitol chewing gum, regular prescribed medications, information about year of birth, and the self-reported outcome of previous oral examination (decayed teeth).

### Statistical Methods

The final number of questions was seven, which were dichotomized for the sum variable analysis. Dichotomization is marked with (a) for each question in every variable in Table II. Restorative treatment need was dichotomized as whether one needed dental restorative treatment (DT > 0) or not (DT = 0). Binary logistic regression models and the Pearson chi-squared test were used to analyze the data in both pretest and final analysis.

All analyses were executed with SPSS software (version 25.0, SPSS, Inc., Chicago, Ill., USA) and Microsoft Excel 16.

### Ethical Considerations

The research plan was evaluated by the Ethical Committee of the Northern Ostrobothnia Hospital District and its positive statement was issued on March 29, 2010. The center for Military Medicine and the Defence Forces Staff gave permission for the study in June 2010 (AG14218/June 23, 2010). All data were anonymized before the analyses.

## RESULTS

Distributions of the responses to the questions between those who continued or interrupted their service are given in Table I. The median year of birth was 1991 (19 years). The younger the conscripts were, the less likely they were to interrupt their service. The conscripts needing regular prescribed medication were few among those who continued their military service compared to almost one in five among those who interrupted their service. Physical exercise in their spare time before the service was very limited among the conscripts who interrupted their military service. In the interruption group, 45.8% did physical exercise less than 1-2 times per month, compared to 20.4% among those who completed the service. Of those who interrupted their service, 22.4% did not work or study before conscription, whereas that percentage was 12.2% in the control group. The group that continued its military service had 10% more of those with no dental restorative treatment need (DT = 0), used xylitol chewing gum more often, and thought that a good state of personal dentition promotes their well-being, unlike those who interrupted their service (Table I).

**TABLE I.** Distribution of Answers to the Final Set Questions among Those Who Continued Military Service and Interrupted Their Military Service

	Continued service	Interrupted service	
	% (*n*)	% (*n*)	*P*
How often do you do physical exercise?			
Never	4.8 (405)	17.4 (46)	<.001^a^
1-2 times a month	15.9 (1,340)	28.4 (75)	
1-2 times a week	37.4 (3,151)	31.4 (83)	
3-4 times a week	30.9 (2,611)	18.2 (48)	
More than five times a week	11.0 (929)	4.5 (12)	
Total (*n*)	100 (8,436)	100 (264)	
What did you do before your service?			
Studied	37.4 (3,160)	27.7 (73)	<.001^a^
Worked	50.4 (4,257)	50.0 (132)	
Took a year off (traveling, etc.)	5.7 (483)	12.9 (34)	
Was unemployed (>6kk)	6.5 (549)	9.5 (25)	
Total (*n*)	100 (8,449)	100 (264)	
Year of birth			
Median age-1 year, (18 years, born 1992)	30.7 (4,109)	15.4 (69)	<.001^a^
Median age starting military service, (19 years, born 1991)	61.6 (8,236)	70.9 (317)	
Median age + 1 year, (20 years, born 1990)	7.7 (1,027)	13.6 (61)	
Total (*n*)	100 (13,372)	100 (447)	
Does your dentition influence your well-being?			
Promotes well-being	46.9 (3,947)	33.3 (88)	<.001^a^
Deteriorates well-being	8.5 (716)	14.8 (39)	
No influence	44.6 (3,751)	51.9 (137)	
Total (*n*)	100 (8,414)	100 (264)	
Restorative treatment need			
DT = 0	55.4 (7,406)	44.5 (199)	<.001^a^
DT > 0	44.6 (5,966)	55.5 (248)	
Total (*n*)	100 (13,372)	100 (447)	
Do you use xylitol chewing gum?			
Yes	88.6 (7,459)	79.9 (211)	<.001^a^
No	11.4 (963)	20.1 (53)	
Total (*n*)	100 (8,422)	100 (264)	
Do you have a chronic illness requiring regular medication?			
Yes	7.5 (635)	18.9 (50)	<.001^a^
No	92.5 (7,801)	81.1 (214)	
Total (*n*)	100 (8,436)	100 (264)	

a Pearson chi-squared test.

In the logistic binary regression model, those who were physically inactive, unemployed, older than 18 years, had regular medication, did not use xylitol chewing gum, thought that state of dentition would not promote their well-being, and needed restorative treatment had an increased risk for interruption of their military service. The area under the receiver-operating characteristic curve was 0.736 (95% confidence interval [CI]: 0.71–0.77) for predicted values. The most prominent single predictive factor for interrupting military service was the amount of weekly exercise. For conscripts who reported having no exercise, the odds ratio (OR) value was 5.80 compared to those who exercised five times per week (Table II).

**TABLE II.** Binary Logistic Regression Model for Each Single Predictive Factor

	Interrupted military service			
	OR	95% CI		*P*
Explanatory factor		Lower	Upper	
How often do you do physical exercise?				
More than five times a week	1			
3-4 times a week	1.39	0.73	2.64	.314
1-2 times a week	1.75	0.95	3.23	.075
1-2 times a month^a^	3.24	1.74	6.04	.000
Never^a^	5.80	3.00	11.22	.000
What did you do before your service?				
Studied	1			
Worked	1.12	0.83	1.52	.447
Took a year off (traveling etc.)^a^	1.67	1.04	2.68	.035
Was unemployed (>6kk)^a^	1.92	1.24	2.97	.004
Year of birth				
Median age—1 year, (18 years, born 1992)	1			
Median age starting military service, (19 years, born 1991)^a^	1.89	1.33	2.70	.000
Median age + 1 year, (20 years, born 1990)^a^	2.83	1.77	4.51	.000
Does your dentition influence your well-being?				
Promotes well-being	1			
Deteriorates well-being^a^	1.81	1.21	2.70	.004
No influence^a^	1.39	1.05	1.84	.021
Restorative treatment need				
DT = 0	1			
DT > 0^a^	1.40	1.08	1.82	.010
Do you use xylitol chewing gum?				
Yes	1			
No^a^	1.42	1.03	1.96	.035
Do you have a chronic illness requiring regular medication?				
No	1			
Yes^a^	2.94	2.12	4.08	.000

a Alternatives marked with (^a^) are chosen to be risk factors (i.e., value 1) in dichotomized sum variable of interrupting military service (Table III).

In the logistic regression model of the sum variable, among those who did not interrupt their military service 50.3% accumulated 0 or 1 predisposing factors, whereas those who interrupted their service 76.5% accumulated more than two predisposing factors. The risk for interruption of military service was 68.6 times higher in cases where six predisposing factors were present compared to those who had none (Table III).

**TABLE III.** Binary Logistic Regression Model for Sum Variable of Predisposing Factors for Interrupting Military Service

					Interrupted military service		
		95% CI			Yes	No	Total
Sum	OR	Lower	Upper	*P*	% (*n*)	% (*n*)	%(*n*)
0	1				3.4 (9)	17.1 (1,441)	16.7 (1,450)
1	3.04	1.49	6.17	0.002	20.1 (53)	33.2 (2,794)	32.8 (2,847)
2	5.18	2.59	10.35	0.000	30.3 (80)	29.4 (2,472)	29.4 (2,552)
3	8.13	4.03	16.41	0.000	24.2 (64)	15.0 (1,260)	15.3 (1,324)
4	18.53	8.94	38.41	0.000	15.9 (42)	4.3 (363)	4.7 (405)
5	27.03	11.21	65.19	0.000	4.9 (13)	0.9 (77)	1.0 (90)
6	68.62	15.27	308.43	0.000	1.1 (3)	0.1 (7)	0.1 (10)
7	–	–	–	–	–	–	–
Total					100 (264)	100 (8,414)	100 (8,678)

## DISCUSSION

The main finding of this study was that, as hypothesized, self-reported physically inactive lifestyle, chronic illness with medication and greater age, as well as unemployment, low perception of one’s own well-being, restorative treatment need, and not using xylitol chewing gum are individually associated with increased risk of interrupting military service. This study showed that well-known predictive factors that are important for general life management also clearly influence the risk of military service interruption. Although these predictive factors are related to general life management and interact with each other, their interactions were not considered in this study, but are widely discussed elsewhere.^14–16^ It is notable that oral health is included among the statistically significant predictive factors, although its effect is not as strong as the others.

Health maintenance, which is also part of general life management, including oral health, showed that the state of oral health can be used as a predictive factor in assessing whether a conscript is at increased risk. The increased need for restorative treatment and the low use of xylitol chewing gum increased the risk of interruption. In Finland, xylitol products are widely used in caries prevention^17^ and recommended by dentists. Xylitol reduces levels of decay-causing *Streptococcus mutans* bacteria in saliva.^18^ Replacing sugar by xylitol in food products may promote better dental health, but evidence is lacking on whether it prevents cavities. Xylitol gums are included in military field ration packages as opportunities to brush one’s teeth are limited, especially in arctic conditions.

The age of a conscript higher than the median was also found to increase the risk of interruption. It may be that the older ones have postponed starting their service for some health reason like serious overweight, which had not completely disappeared by the time of starting conscription. Obeying the rules and regulations is perhaps easier for the younger ones, but this was not factored in here. Economic reasons can also increase the risk of interruption, even though Finland has good financial support systems for those in military service.^19^

The study also examined how the accumulation of predictive risk factors affects the overall risk of interruption of military service. In the sum variable of predictive risk factors, a conscript who responded negatively to general life management or to oral health questions alone exhibited more than eight times the risk of interruption. If greater age was taken into account, the risk increased to 18.5 fold. The results indicate that general life management skills are weaker in the group who end up interrupting their service. An increase in sum variable points steadily increases the likelihood of interrupting service, with six sum variable points reaching an OR of 68.62. This result was derived from a sample of three conscripts who interrupted their service and seven conscripts who completed their military service (95% CI: 15.27, 308.43).

In Finland, mandatory military service provides an exceptional opportunity for conducting epidemiological studies. The study sample comprehensively represents the corresponding Finnish age and male cohort. In this study, the data collection and all the examinations were conducted during the first 2 weeks of military service, which minimized the number of dropouts in the study.

The study groups were chosen according to the outcome (interruption or completion). Another way to form the study groups within the same population would have been to compare those who interrupted their military service with those who decided to apply for civilian service as Lehesjoki did in his study.^3^

Data on those interrupting their military service was previously published by Lehesjoki in 2018. Study groups C and D here were included both in Lehesjoki’s thesis^3^ and in this study. Despite the control group differing from Lehesjoki’s in being much larger, the results were similar. Lehesjoki found that smoking and alcohol consumption were statistically significant predisposing factors for interruption. Smoking and increased alcohol consumption was also found as a predictive factor for interruption among Lithuanian conscripts.^20^ The same variables were also investigated in this study in pretest phase but were ultimately found to be only marginally significant. This difference could be explained by differences in the sizes of the control groups or determining the amount of alcohol used and smoking.

The results of both studies have similar features. Lehesjoki found that regular medication is a factor for interruption and proposed that a large portion of those with a respiratory-disease-based interruption (10.2%) consist mainly of asthma and its medication. Lehesjoki also found that mental and behavioral disorders (30.8%) were a predisposing factor for interruption. Also, in study of Lithuanian conscripts, those who completed their military service were physically more active and had less psychological distress.^20^ In turn, medication for mental and behavioral disorders and asthma can be considered as a predisposing factor for dental caries,^21^ which was found to be a predisposing factor for interruption in this study. A complete comparison of the results between Lehesjoki and this study is impossible because of interdependent cases and differently sized control groups. However, in Finland, military service is the last mandatory health checkpoint for the Finnish male population. Identifying the health-related reasons for the interruption of military service could be used to develop means of health and social support after the interruption of military service. These means may also help to prevent early disability retirement later in life.^22^

Both studies conducted the general and oral examinations and questionnaires of conscripts during the first three weeks. In the five biggest garrisons random sampling was conducted by selecting every fifth draftee in alphabetical order to oral examination, as the result of a limited timetable and a lack of resources. The limited time window and the time reserved for the oral examinations meant that some of the conscripts did not have enough time to answer the questionnaire. These limitations and the cross-sectional nature of this study can be considered as major weaknesses. On the other hand, oral examined conscripts comprised over 50% of all annual recruits of the year 2011 in Finland, which can be considered as a representative study sample. Also, all conscripts who interrupted their service and underwent oral examination represent 15.4% of the entire annual interruption group in Finland in the year 2011. Finnish conscripts comprise a very unique study sample. They represent very well major age groups (18, 19, and 20 year olds) every year regardless of their socioeconomic, demographic, or geographic background. From this perspective study, samples can be considered as strength of the study.

## CONCLUSION

The results show that the set of statistically selected questions could help recognize conscripts who have an increased risk of interruption of military service. Although the set of questions was applicable to the present study cohort, further implications should be made with caution. Further studies are needed; for example, the questions should be validated before any further use for different populations. The recognition of those with a bigger risk of interrupting military service could be worth paying special attention to.

## References

[1] Finnish Defence Forces : First to call-ups. Available at https://maavoimat.fi/en/-/kutsuntojen-kautta-palvelukseen; accessed March 11, 2021.

[2] Statistics Finland : Conscription. Available at https://findikaattori.fi/fi/99; accessed March 11, 2021.

[3] Lehesjoki M : Interruption of Conscript Service for Health Reasons. National Defence College, Publication Series 1: Studies No. 27, 19992018. National Defence University, PhD thesis, Helsinki, 2018. Available at https://www.doria.fi/handle/10024/157248; accessed March 11, 2021.

[4] Sahi T , Korpela H: *Interruption of Military Service Due to Health Reasons in 1997 – 2000*. Publications of the Department of Military Medicine; 2002, pp 4–14.

[5] Ankkuriniemi O : The Dental Status and Need for Dental Treatment among Finnish Conscripts. University of Helsinki, PhD thesis, Helsinki, 1979. 7443698

[6] Läärä M : Polarization of Dental Caries and the Explanatory Background Facts in a Finnish Conscript Population. University of Turku, PhD thesis, Turku, 1999.

[7] Kämppi A : Identifying Dental Restorative Treatment Need in Healthy Young Adults at Individual and Population Level. University of Oulu, PhD thesis, Oulu, 2015. Available at http://urn.fi/urn:isbn:9789526209128; accessed March 11, 2021.

[8] Tanner T : Healthy Young Adults’ Oral Health and Associated Factors: Cross-Sectional Epidemiological Study. University of Oulu, PhD thesis, Oulu, 2015. Available at http://urn.fi/urn:isbn:9789526208558; accessed March 11, 2021.

[9] Tanner T , Kämppi A, Päkkilä J, et al: Prevalence and polarization of dental caries among young, healthy adults: cross-sectional epidemiological study. Acta Odontol Scand 2013; 71(6): 1436–42. 23627898 10.3109/00016357.2013.767932

[10] Kämppi A , Tanner T, Päkkilä J, et al: Geographical distribution of dental caries prevalence and associated factors in young adults in Finland. Caries Res 2013; 47(4): 346–54. 23548873 10.1159/000346435

[11] World Health Organization : Oral Healt Surveys. Available at http://www.icd.org/content/publications/WHO-Oral-Health-Surveys-Basic-Methods-5th-Edition-2013.pdf; accessed March 11, 2021.

[12] Kämppi A , Tanner T, Päkkilä J, et al: Comparison of simple screening criteria with the International Caries Detection and Assessment System classification in determining restorative treatment need. Int Dent J 2016; 66(2): 63–70. 26503398 10.1111/idj.12204 PMC9376493

[13] Anttonen V , Seppä L, Niinimaa A, et al: Dietary and oral hygiene intervention in secondary school pupils. Int J Paediatr Dent 2011; 21(2): 81–8. 20731735 10.1111/j.1365-263X.2010.01095.x

[14] Current Care Guidelines : Exercise. Available at https://www.kaypahoito.fi/hoi50075; accessed March 11, 2021.

[15] Mousteri V , Daly M, Delaney L, et al: Adolescent mental health and unemployment over the lifespan: population evidence from Sweden. Soc Sci Med 2019; 222: 305–14. doi: 10.1016/j.socscimed.2018.12.030 30677644

[16] Ali SM , Lindström M: Psychosocial work conditions, unemployment, and leisure-time physical activity: a population-based study. Scand J Public Health 2006; 34(2): 209–16. 16581714 10.1080/14034940500307515

[17] Current Care Guidelines : Effect of regular use of xylitol chewing gum or lozenge on tooth decay and on the onset and progression of early caries. Available at https://www.kaypahoito.fi/nak06746; accessed March 11, 2021.

[18] Eby A , Ankola A: Effectiveness of xylitol and polyol chewing gum on salivary streptococcus mutans in children: a randomized controlled trial. Indian J Dent Res 2018; 29(4): 445–9. 30127194 10.4103/ijdr.IJDR_307_16

[19] KELA : Available at https://www.kela.fi/web/en/conscripts-allowance?inheritRedirect=true; accessed July 21, 2021.

[20] Mieziene B , Emeljanovas A, Cesnaitiene VJ, et al: Health behaviors and psychological distress among conscripts of the Lithuanian military service: a nationally representative cross-sectional study. IJERPH 2020; 17(3): 783. 10.3390/ijerph17030783 PMC7037156 32012683

[21] Agostini BA , Collares KF, Costa FDS, et al: The role of asthma in caries occurrence - meta-analysis and meta-regression. J Asthma 2019; 56(8): 841–52. 29972654 10.1080/02770903.2018.1493602

[22] Heikki Frilander H , Lallukka T, Viikari-Juntura E, et al: Health problems during compulsory military service predict disability retirement: a register-based study on secular trends during 40 years of follow-up. PLoS One 2016; 11(8): e0159786. 10.1371/journal.pone.0159786 PMC4988709 27533052

